# Aging-modulating treatments: from reductionism to a system-oriented perspective

**DOI:** 10.3389/fgene.2014.00446

**Published:** 2014-12-19

**Authors:** Alexander M. Vaiserman

**Affiliations:** Laboratory of Epigenetics, D. F. Chebotarev State Institute of Gerontology NAMS of UkraineKiev, Ukraine

**Keywords:** anti-aging drugs, antioxidants, geroprotectors, hormesis, life extension

The delay of aging and healthy life extension is a primary concern of modern gerontological research. Based on theoretical assumptions and empirical findings, a number of bioactive agents have been proposed as promising “anti-aging” (geroprotective) preparations (de Magalhães et al., 2012; Kennedy and Pennypacker, 2014). Usually, the substance is considered to have aging-modulating activity if its efficiency can be confirmed both *in vitro* and *in vivo*. In the first step of screening, a hypothetical geroprotective compound (e.g., antioxidant known to play a key role in ROS scavenging) is examined in *in vitro* models. If its effectiveness is confirmed, then its aging-modulating properties are studied in experimental *in vivo* models. In the case where supplementation of experimental animals with this agent causes life extension, it advertised as anti-aging drug. A logic discrepancy, however, can be generated between these two steps. Indeed, even if some substance is highly effective in *in vitro* model due to influencing a specific age-related pathway, its life-extending effects in animal models might be caused by unspecific (hormetic) mechanisms.

Hormetic responses are commonly referred to as stimulatory effects occurring in response to low levels of exposure to stressors or agents that are harmful at high levels of exposure. The prevailing hypothesis explaining the hormetic phenomenon is that a low dose of a toxic agent induces damage; following this damage, a repair response is initiated which result in a slight overcompensation to the disruption of homeostasis, i.e., a general response of the organism (Calabrese, 2013). According to this hypothesis, various mild stresses and low-dose pharmacological treatments may cause rather similar beneficial effects because these effects are not mediated by specific receptors. There is a large corpus of data demonstrating that hormesis is implicated in health and disease across various species including human beings (Rattan and Le Bourg, 2014). Longevity hormesis was repeatedly obtained for a number of obviously harmful substances including herbicides, pesticides, heavy metals, and hydrocarbons (Neafsey, 1990). The hormetic model of dose response is usually described as U-shaped curve ranging from impairment at levels of deficient intake, to optimal function at intermediate levels, and to toxicity at excessive intake levels (Calabrese, 2013). All prospective aging-modulating agents are essential nutrients necessary to support health and well-being. These dietary components including minerals, trace elements, vitamins and antioxidants, however, similarly to hormetic compounds (“hormetins”) all exhibit toxic effects at excess levels, i.e., they show typical hormetic dose response (Rattan, 2008). Indeed, while the moderate daily levels of vitamins and minerals are both required and beneficial, their excessive dietary levels are known to cause hypervitaminosis, tissue mineralization, or electrolyte imbalance (Hayes, 2007). The excessive intake of antioxidants and hormones is known to destroy delicate control mechanisms of homeodynamics, and it seems therefore unlikely that they have a long-term beneficial impact (Goto, 2004).

Furthermore, both presumably aging-modulating effects and hormetic effects do not preclude the coexistence of toxicity and other adverse outcomes. The predominance of beneficial (life-extending) or deleterious effects may vary not only with dose but also with species, gender, age, stage of life, disease or disability (Minois, 2000). In some cases, life extension caused by both hormetins and geroprotectors may be accompanied by side effects such as impairment of reproductive and immune functions, as well as stress resistance (Matsagas et al., 2009; Saul et al., 2013; McClure et al., 2014). In addition, pharmaceutical interventions can act as caloric restriction (CR) mimetics due to the suppression of the appetite, and their life-extending effects may be caused, at least partially, by the CR-inducing hormetic response (Masoro, 2007).

Given all these consideration, it can be concluded that similarity may exist in the mode of action between hormetins and geroprotectors. Currently, however, these are no valid criteria to distinguish mechanistic pathways between them. Such criteria were first proposed and efforts were made to validate them as early as in the 60–70 s of the last century in works of George A. Sacher which distinguished the life-extending effects of substances acting by a “proper action” (a specific role of these agents in reducing the accumulation of age-associated lesions or protecting against age-related disorders) from the hormetic (non-specific) effects (Sacher, 1977). According to this point of view, a hormetic response is a function of the state of an organism, rather than a kind of the stimulant. Furthermore, it has been suggested that such effect could only be observed in depressed or ill individuals and, therefore, hormesis would not result in an increase of maximal life span but would serve only to enable the animal to approach its potential longevity due to nullification of several adverse environmental effects (Sacher and Trucco, 1962). In addition, Sacher and Trucco postulated that hormesis can be found only if control animals are short-lived. In subsequent studies, however, it became clear that efficiency of geroprotectors is also strongly dependent on the viability of control population (Izmaylov and Obukhova, 1996). A tendency to increase the mean life span rather than the maximal life span was similarly found for both hormetic (Goto, 2004) and geroprotective (Bonnefoy et al., 2002; Spindler, 2003) actions. Moreover, the magnitude of the life-extending effects of geroprotectors is consistent with that seen for hormetins. In most cases, the maximum magnitude of such effects does not exceed 20–30% (Frolkis and Muradian, 1991; Olshansky et al., 2002; Spindler, 2003).

Sacher also assumed that hormetically-induced life extension is distinct from that caused by “proper actions” in the shape of age-specific logarithmic mortality curves and their Gompertz approximations (the exponential equation to approximate the probability of death as a function of age). For example, low-dose radiation exposure, according to his point of view, leads to decrease of the Gompertz intercept (frailty), while caloric restriction (“proper action,” by Sacher) causes a decrease of the Gompertz slope (rate of senescence) (Sacher, 1977). More recent studies, however, were unable to confirm such assumption. For example, Doubal and Klemera (1999) by analyzing survival data from many studies revealed that the effects of caloric restriction as well as antioxidants on the behavior of mortality curves in mice and rats do not indicate that these treatments alter the rates of aging.

In summarizing these findings, it becomes obvious that Sacher's criteria of mechanistic demarcation between the hormetically-induced and “proper action”-induced life-extending effects are likely doubtful. Indeed, the efficiency of anti-aging drugs is generally attributed to their specific geroprotective activities. This explanation, however, ignores the fact that aging process, rather than changes in individual cells, tissues, or organs, may be more a function of the deterioration of integrative mechanisms, for instance, in the central nervous system (Shock, 1977). Since aging process is characterized by increased homeodynamic imbalance and progressive impairment of organism's adaptive abilities, the hormetic stimulation of the organism's maintenance and repair pathways known to be regulated by integrative mechanisms (Minois, 2000), may induce the body's own ability to combat stresses, including aging. Thus, although the potential aging-modulating drugs can really affect some specific molecular and cellular pathways related to aging, their life-prolonging effects may likely be explained by unspecific hormetic responses. It can be hypothesized that certain compound can cause life-extending effect because it was applied within the optimal hormetic response zone for this substance. At the same time, when used in high dose, it may exhibit adverse effects similarly to all conventional toxicants. Indeed, U-shaped (hormetic-like) dose–response relationship for health benefits has been repeatedly demonstrated for a variety of prospective aging-modulating compounds. For example, in a variety of clinical trials it has been shown that high doses of antioxidant supplements may be associated with unwanted consequences for the health and higher all-cause mortality, especially in well-nourished populations (Calabrese et al., 2010; Dolara et al., 2012; Goyal et al., 2013; Bjelakovic et al., 2014).

In the consideration of life-extending effects of aging-modulating drugs, a logical error can occur as a result of reductionist thinking peculiar to gerontologists in the middle of the last century when major aging theories including the free radical theory of aging were proposed. From the reductionist point of view, the organism was considered as a sum of relatively independent processes and mechanical components, and interventions designed to prolong life were seen as those being similar to car repairing. If this were indeed the case, then it would be possible to slow the rate of aging by affecting molecular pathways that influence specific aspects of aging, analogous to how antioxidants can slow down the rate of aging of plastic. However, by summarizing the accumulated information, one can conclude that a reductionist approach in experimental gerontology has proved rather ineffective until now. This is not surprising, since aging is a classic “complex trait,” in other words, a trait that is influenced by a plurality of genetic pathways. For example, Rose and Burke (2011) reported, based on their own genome-wide research in *Drosophila* and findings by other authors, that hundreds of genes are involved in the control of aging. Therefore, it seems very difficult, if not impossible, to develop effective pharmaceutical interventions that may slow aging and extend longevity by targeting single genetic pathways.

On the contrary, more modern systemic (“holistic”) thinking considers the organism as a whole (Fardet and Rock, 2014). Taking into account the complexity of the aging process, the systemic approach addressed primarily to central regulation mechanisms seems more appropriate to developing aging-modulating treatments. From a systemic point of view, aging is not a disease in the sense of being caused by disturbance in several specific pathway(s), but is rather an inevitable consequence of realization of some (probably still substantially unknown) central regulatory processes making the organism more vulnerable to disease with age. According to these conceptual frameworks, aging process is not primarily a result of accumulation of stochastic damage but is rather a co-product of developmentally regulated processes (de Magalhães, 2012).

One potential mechanism of central regulation of the whole life cycle including aging is a process of epigenetic control of gene expression having important features in the given context. Indeed, it is: (1) potentially adaptive; (2) linking development and aging; (3) generalizing at the whole-organism level. It is noteworthy that modulation of epigenetic processes may amazingly influence the longevity. For example, in social insects queens and workers sometimes exhibit a 100-fold difference in longevity, with reproductive queens having much longer lifespan than non-reproductive workers. There is emerging evidence that extremely long-lived queen phenotype is driven by epigenetic mechanisms of gene regulation (Welch and Lister, 2014). Epigenetic mechanisms are also likely involved in the hormetic phenomenon (Vaiserman, 2011), and treatment with epigenetic drugs, e.g., pharmacological inhibitors of histone deacetylases, can lead to a significant life extension in model organisms (Vaiserman and Pasyukova, 2012).

In conclusion, the transition from reductionism to a system-oriented perspective (Russo et al., 2014) and utilizing the systematic approaches in modern biogerontology may likely result in developing novel aging-modulating treatment strategies and provide new ways to extend human health span.

## Conflict of interest statement

The author declares that the research was conducted in the absence of any commercial or financial relationships that could be construed as a potential conflict of interest.
